# Downregulation of microRNA‐6125 promotes colorectal cancer growth through YTHDF2‐dependent recognition of N6‐methyladenosine‐modified GSK3β

**DOI:** 10.1002/ctm2.602

**Published:** 2021-10-14

**Authors:** Hongyan Li, Ning Zhang, Xueli Jiao, Cong Wang, Wenhao Sun, Yanyu He, Ganglin Ren, Shirui Huang, Mengjie Li, Yixin Chang, Zihui Jin, Qipeng Xie, Xiaodong Zhang, Haishan Huang, Honglei Jin

**Affiliations:** ^1^ Zhejiang Provincial Key Laboratory of Medical Genetics Key Laboratory of Laboratory Medicine Ministry of Education School of Laboratory Medicine and Life Sciences Wenzhou Medical University Wenzhou China; ^2^ The Second Affiliated Hospital & Yuying Children's Hospital of Wenzhou Medical University Wenzhou China; ^3^ The First Affiliated Hospital of Wenzhou Medical University Wenzhou China

**Keywords:** cell cycle, colorectal cancer, GSK3β, m6A, miR‐6125, Wnt/β‐catenin, YTHDF2

## Abstract

**Background:**

MicroRNAs (miRNAs), the key regulator of gene expression, and N6‐methyladenosine (m6A) RNA modification play a significant role in tumour progression. However, regulation of m6A‐modified mRNAs by miRNAs in colorectal cancer (CRC), and its effect on progression of CRC, remains to be investigated.

**Methods:**

Expression of miR‐6125 and YTH Domain‐Containing Family Protein 2 (YTHDF2) was detected by western blotting and immunohistochemistry. The effects of miR‐6125 and YTHDF2 on proliferative capacity of CRC cells were analysed using soft agar, ATP, CCK8 and EdU assays, and in animal experiments.

**Results:**

MiR‐6125 expression was downregulated markedly in CRC, and expression correlated negatively with tumour size and prognosis. MiR‐6125 targeted the 3′‐UTR of *YTHDF2* and downregulated the YTHDF2 protein, thereby increasing the stability of m6A‐modified glycogen synthase kinase 3 beta (*GSK3β*) mRNA. Increased GSK3β protein levels inhibited the expression of Wnt/β‐catenin/Cyclin D1 pathway‐related proteins, leading to G0‐G1 phase arrest and ultimately inhibiting the proliferation of CRC cells.

**Conclusions:**

MiR‐6125 regulates YTHDF2 and thus plays a critical role in regulating the Wnt/β‐catenin pathway, thereby affecting the growth of CRC. Collectively, these results suggest that miR‐6125 and YTHDF2 are potential targets for treatment of CRC.

## Graphical abstract

MiR‐6125 regulates YTHDF2 and thus plays a key role in regulating the Wnt/β‐catenin pathway, thereby affecting growth of CRC both in vitro and in vivo. The results suggest that miR‐6125 and YTHDF2 are potential targets for treatment of CRC.

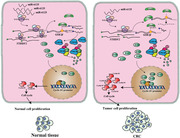

## 1 INTRODUCTION

Colorectal cancer (CRC) is one of the most common cancers worldwide, and its incidence and mortality rates are increasing.^1^ More than 1.8 million new cases of CRC, and more than 860 000 deaths, were reported in 2018, and the global burden of CRC continues to increase.^2^ Targeted therapy is one of the most effective treatments, and various targeted drugs are currently available.^3^, ^4^, ^5^ Despite considerable advances in the treatment of CRC, the overall clinical results are not satisfactory. Identifying new targets and developing effective drugs against them are important objectives for clinical and basic research.

MicroRNAs (miRNAs), belonging to a kind of non‐coding RNA, with a length of about 20–24 nucleotides, are encoded by endogenous genes and expressed widely in different species and tissues.^6^ MiRNAs have attracted extensive research attention because of their important role in the progression of cancer.^7^, ^8^, ^9^, ^10^, ^11^ Although they play key roles in the progression of CRC, the functions and underlying mechanisms of many miRNAs remain to be elucidated, limiting our understanding of the progression of CRC as well as the development and application of related drugs. MiR‐6125 is located on chr12q14.1,^12^ and its biological function and mechanism of action remain unknown. Recently, we first demonstrated that miR‐6125 is downregulated significantly in human CRC, and that it inhibits the progression of CRC by downregulating *YTHDF2* expression.

N6‐methyladenosine (m6A) modification is the most common RNA modification in higher organisms; it regulates the cleavage, transport, localization, stability and translation of RNA at the post‐transcriptional level.^13^, ^14^, ^15^, ^16^, ^17^ Indeed, m6A modification is involved in many biological processes, including tumourigenesis.^18^ YTHDF2 is an m6A reader protein that recognizes and degrades RNA modified by m6A modification.^19^ YTHDF2 plays different roles in various cancers, it can promote as well as inhibit tumour progression, playing an important and versatile role.^20^, ^21^, ^22^, ^23^ Despite its known role in tumour progression, the expression, regulation and specific biological function of YTHDF2 in CRC remain unclear.

The Wnt signalling pathway is an important regulatory pathway with multiple links and multiple action sites; the pathway is necessary for embryo growth and development. Abnormal activation of this pathway is associated with the progression of multiple cancers.^24^ Approximately 90% of CRC cases are related to abnormal activation of the Wnt pathway;^25^ therefore, targeting the Wnt pathway is an effective treatment for CRC. However, Wnt signalling is regulated by complex mechanisms, and a comprehensive understanding of these mechanisms remains elusive.

In this study, we showed that YTHDF2 recognizes and targets m6A‐modified GSK3β mRNA for degradation. This decreases the level of p‐β‐catenin, thereby inhibiting ubiquitination and degradation of β‐catenin and enhancing the stability of the β‐catenin protein. β‐Catenin thus accumulates in the nucleus and promotes the transcription of its downstream gene Cyclin D1. The present results provide a detailed understanding of the relationship between miRNAs, RNA methylation, the Wnt/β‐catenin pathway and CRC progression, and identify miR‐6125 and YTHDF2 as potential targets for the clinical treatment of CRC.

## 2 RESULTS

### 2.1 MiR‐6125 is downregulated in CRC cell lines and CRC tissues, and is correlated with tumour size and prognosis in CRC patients

**FIGURE 1:**
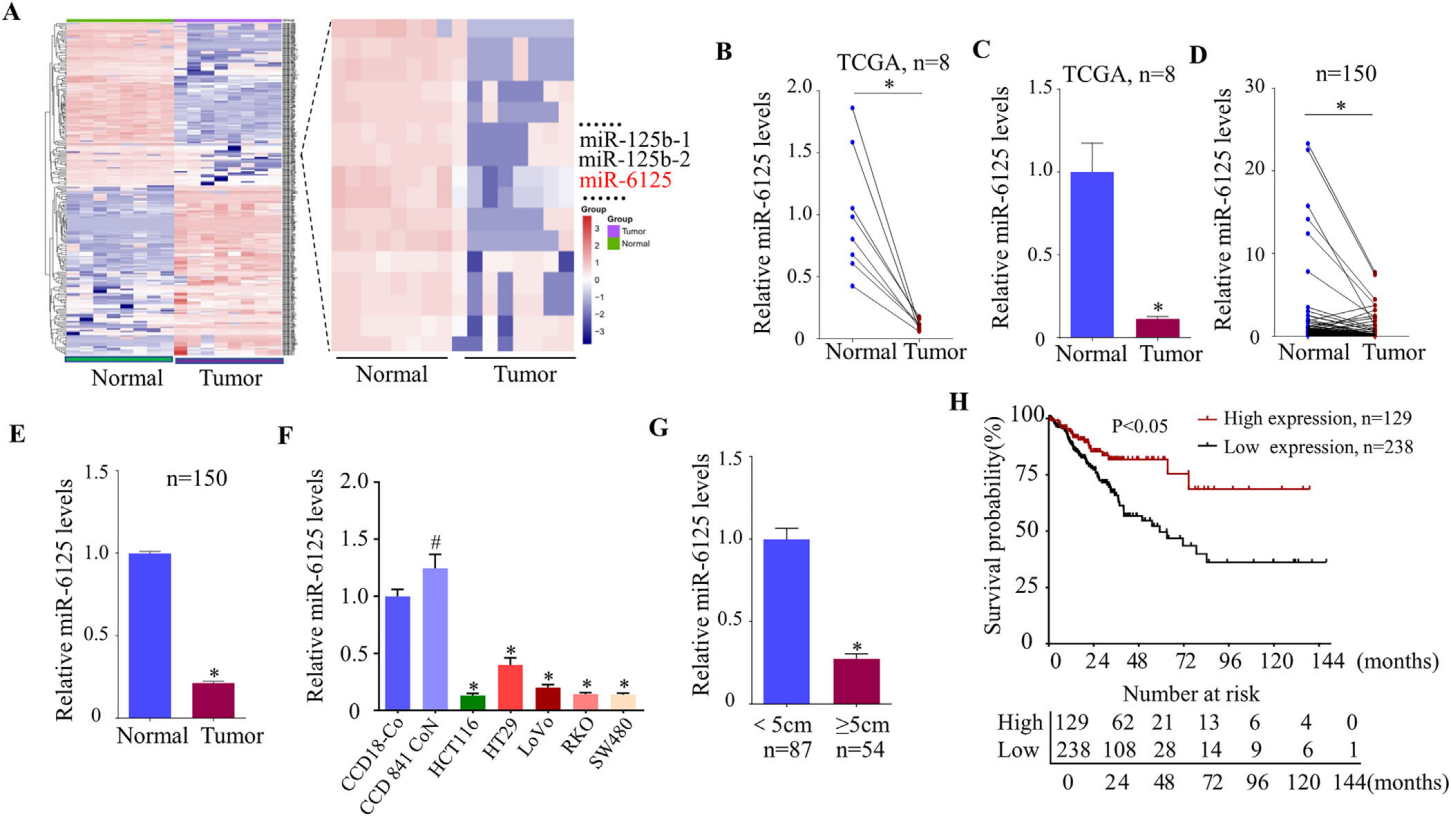
MiR‐6125 is downregulated in human CRC tissues and cell lines and is associated with prognosis in patients. (A) Heat map of the expression of miRNAs in CRC and paired adjacent normal tissues from the TCGA database. (B and C) Differential expression of miR‐6125 in CRC and paired adjacent normal tissues from the TCGA database (*n* = 8). (D and E) QPCR analysis of the expression of miR‐6125 in matched CRC tissues (*n* = 150). (F) QPCR analysis of miR‐6125 expression in normal colorectal epithelial cells (CCD‐18 Co and CCD 841 CoN) and CRC cells (HCT116, HT29, LoVo, RKO and SW480). (G) QPCR analysis of the expression of miR‐6125 in colorectal tumours with a largest diameter of < 5 cm (*n* = 87) and ≥5 cm (*n* = 54). (H) The relationship between miR‐6125 expression data from the TCGA database and disease‐free survival was analysed using survival curves. An asterisk (*) indicates a significant difference at *p *< 0.05

To identify miRNAs involved in CRC process, we explored the sequencing data from The Cancer Genome Atlas (TCGA) database (Figure 1A). Analysis of the eight pairs of adjacent normal tissues (ANTs) and CRC tissues indicated that miR‐6125 was downregulated significantly in CRC tissues (Figure 1B and C). Analysis of 150 pairs of fresh CRC clinical samples (the basic information of clinical samples was shown in our previous study^26^) by qRT‐PCR showed that miR‐6125 expression level was lower in CRC tumour tissues than in ANTs (Figure 1D and E), which was consistent with the expression of miR‐6125 in TCGA database. In addition, miR‐6125 expression level was lower in the CRC cells (HCT116, HT29, LoVo, RKO and SW480) than in the normal colon cells (CCD 841 CoN and CCD‐18Co) (Figure 1F). The effect of miR‐6125 on progression of CRC was examined by dividing CRC tissues into a < 5 cm group (*n* = 87) and a ≥5 cm group (*n* = 54) according to the largest diameter of the colorectal tumour (the information of 9 cases in all of 150 clinical cases was not available, more details are described in Table S1). Expression level of miR‐6125 was notably downregulated in the ≥5 cm group compared to the < 5 cm group (Figure 1G), indicating that miR‐6125 may be related to tumour growth. Analysis of 367 CRC patients from the TCGA database showed that low miR‐6125 levels were associated with a worse prognosis (Figure 1H). Collectively, these results showed that miR‐6125 maybe critic in progression of CRC, and may serve as a diagnostic and prognostic marker.

### 2.2 MiR‐6125 significantly suppresses the proliferation capacity of CRC cells in vitro and in vivo

**FIGURE 2:**
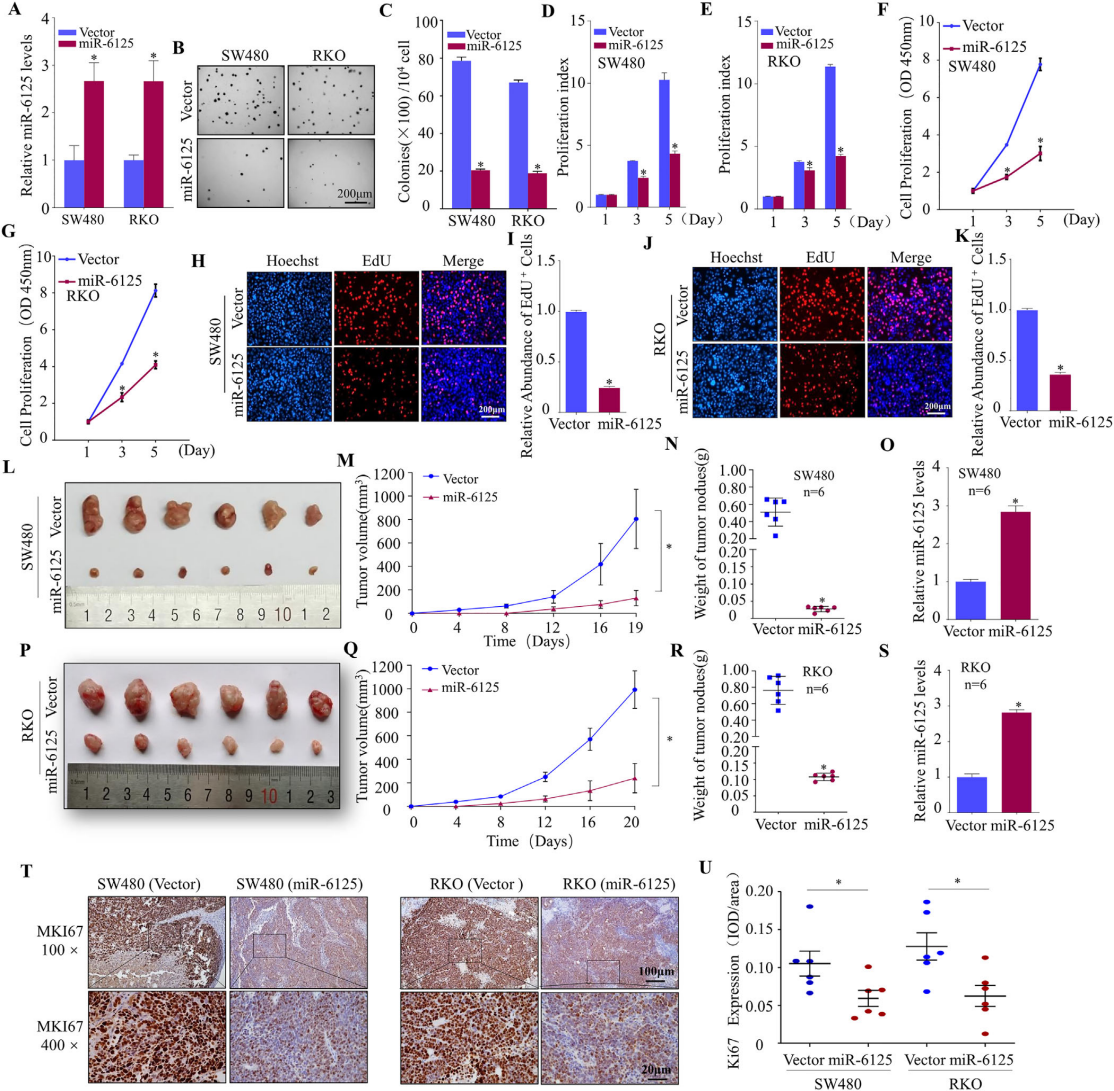
MiR‐6125 inhibits the proliferation of CRC in vitro and in vivo. (A) MiR‐6125 and its control vector plasmid were transfected into SW480 and RKO cells to obtain stable transfected cells; transfection efficiency was detected by qPCR. (B and C) The effect of miR‐6125 overexpression on the anchorage‐independent growth of SW480 and RKO cells was analysed in a soft agar assay. (D and E) Effect of miR‐6125 on the proliferation rate of SW480 and RKO cells, as analysed by the ATP assay. (F and G) Effect of miR‐6125 on the proliferation rate of SW480 and RKO cells, as detected by the CCK8 assay. (H–K) EdU assay to examine the effect of miR‐6125 on the DNA replication activity of SW480 and RKO cells. (L–S) Nude mice were injected subcutaneously with SW480 (miR‐6125) or RKO (miR‐6125) and the corresponding control stable cell lines. After about 3 weeks, subcutaneous tumours were excised and photographed. A growth curve was drawn, the tumours were weighed, and miR‐6125 expression in the tumour was detected by qPCR. (T and U) Tumour samples were fixed and stained with haematoxylin and eosin. Differences in MKI67 expression were detected by immunohistochemical staining. An asterisk (*) indicates a significant difference at *p* < 0.05

To investigate whether the downregulation of miR‐6125 in CRC is of great significance to the CRC progression, we selected SW480 and RKO cell lines with relatively low expression of miR‐6125 for subsequent functional and mechanism studies. Cell lines SW480 (miR‐6125) and RKO (miR‐6125) stably expressing miR‐6125, and their respective control cell lines SW480 (Vector) and RKO (Vector), were constructed and verified by qPCR (Figure 2A). Then, the proliferation of miR‐6125‐expressing CRC cell lines was tested in vitro. Soft agar assays indicated that miR‐6125 inhibited the colony‐forming ability of CRC cells significantly (Figure 2B and C). The ATP and CCK8 assays confirmed that miR‐6125 inhibited the proliferation of CRC cells (Figure 2D–G). The EdU assay demonstrated that miR‐6125 inhibited the DNA replication activity of CRC cells significantly (Figure 2H–K). In order to study the effect of miR‐6125 on proliferative capacity of CRC cells in vivo, a nude mouse xenograft tumour model was established. The results showed that stable overexpression of miR‐6125 (Figure 2O and S) inhibited the size, volume and weight of subcutaneous tumours in nude mice significantly compared with control group (Figure 2L–N and P–R). Immunohistochemistry (IHC) staining of mouse tissues showed that overexpression of miR‐6125 significantly decreased the positivity rate of the proliferation marker MKI67 (Figure 2T and U). The above results indicated that miR‐6125 may act as a CRC suppressor gene to inhibit proliferative capacity of CRC cells in vitro and in vivo.

### 2.3 YTHDF2 is the direct target gene of miR‐6125 in CRC cells and functions as an oncogene

**FIGURE 3:**
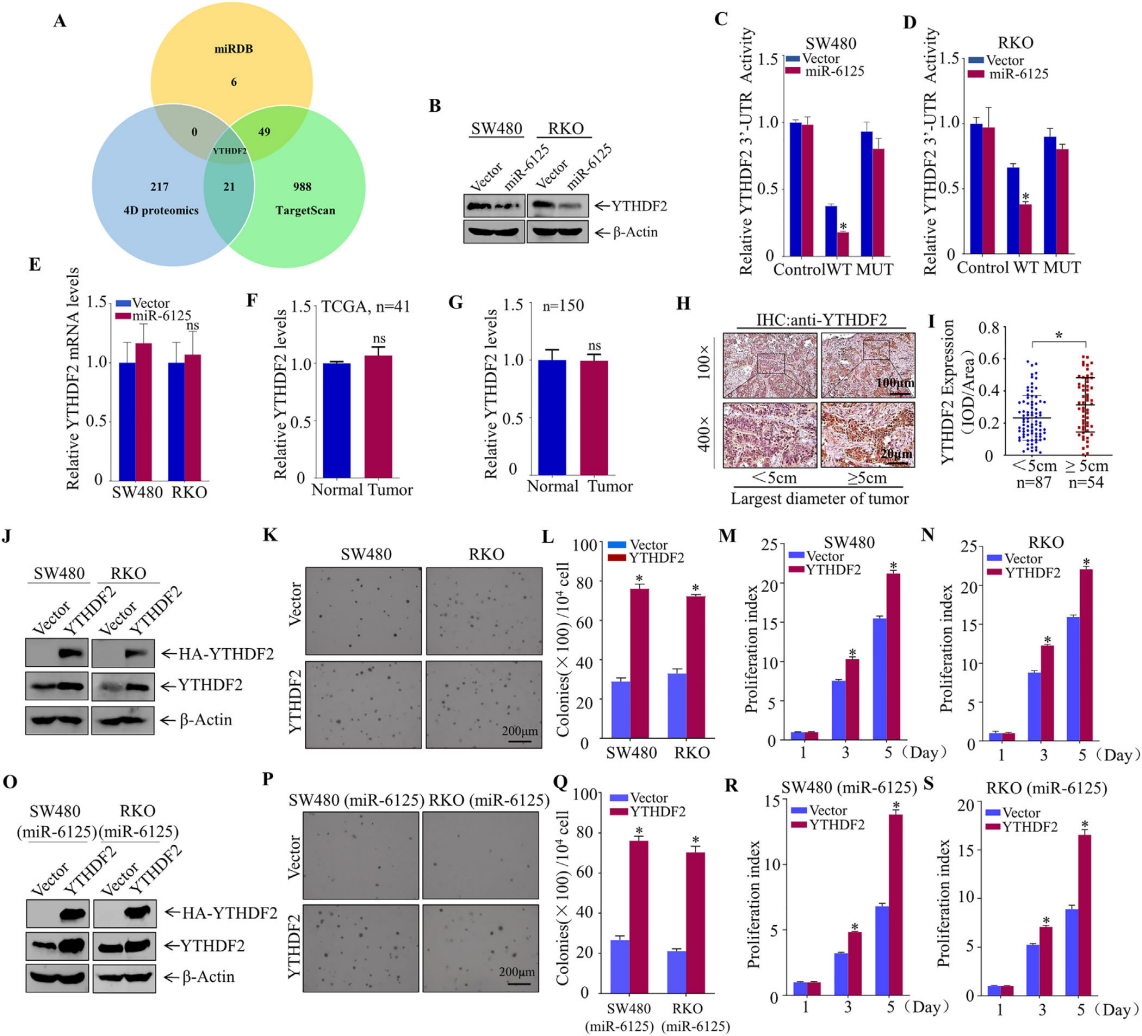
YTHDF2 is the direct target of miR‐6125 and it acts as an oncogene in CRC. (A) TargetScan, miRDB software and 4D proteomics were used to identify the target of miR‐6125. (B) Western blot analysis of YTHDF2 expression in SW480 and RKO cells overexpressing miR‐6125. (C and D) *YTHDF2* 3′‐UTR wild‐type, mutant and control plasmids were transiently transfected into SW480 (vector), SW480 (miR‐6125), RKO (vector) and RKO (miR‐6125) cells, and the dual luciferase activity of the transfected cells was measured. (E) *YTHDF2* mRNA expression levels in SW480 and RKO cells stably overexpressing miR‐6125 were detected by qPCR. (F) The expression of *YTHDF2* in 41 paired tissues was analysed in TCGA database. (G) QPCR analysis of fresh 150 CRC and paired normal tissue samples. (H and I) IHC staining of 150 pairs of freshly collected CRC tumour and adjacent normal tissues. CRC tumours were divided into two groups according to the largest diameter (< 5 cm group, *n* = 87; ≥5 cm group, *n* = 54), and YTHDF2 expression was compared. (J and O) YTHDF2‐overexpression and control plasmid vectors were stably transfected into SW480, RKO, SW480 (miR‐6125) and RKO (miR‐6125) cells. Transfection efficiency was detected by western blotting. (K–N) Effect of YTHDF2 on anchorage‐independent growth and proliferation of SW480 and RKO cells was detected by soft agar cloning and ATP assays. (P–S) Effect of YTHDF2 on anchorage‐independent growth and proliferation in SW480 (miR‐6125) and RKO (miR‐6125) cells was detected by soft agar and ATP assays. An asterisk (*) indicates a significant difference at *p* < 0.05

MiRNAs generally play biological roles by binding to the 3′‐untranslated region (3′‐UTR) of a target mRNA and regulate its expression.^27^ Here, we used bioinformatics software (TagetScan and miRDB) to analyse the potential target genes of miR‐6125, and analysed the proteins significantly downregulated in SW480 (miR‐6125) compared with SW480 (vector) cells through 4D proteomics. After the intersection of the three, it is found that YTHDF2 is the only intersection gene (Figure 3A). YTHDF2 expression in SW480 (miR‐6125) and RKO (miR‐6125) cells was significantly downregulated compared with SW480 (vector) and RKO (vector) cells using western blot assay (Figure 3B). A dual‐fluorescence experiment was then performed to determine whether miR‐6125 regulates *YTHDF2* by targeting the 3′‐UTR directly. For this purpose, wild‐type and mutant 3′‐UTR constructs were generated (Figure S1A). *YTHDF2*‐3′‐UTR WT and *YTHDF2*‐3′‐UTR MUT fluorescein reporters were transfected into SW480 (Vector), RKO (Vector), SW480 (miR‐6125) and RKO (miR‐6125) cells. Thymidine kinase was used as an internal control. The results showed that miR‐6125 overexpression inhibited the activity of *YTHDF2*‐3′‐UTR WT significantly, whereas it had no effect on the activity of *YTHDF2*‐3′‐UTR in the PGL3‐control group or *YTHDF2*‐3′‐UTR MUT (Figure 3C and D). These results indicate that miR‐6125 targets the 3′‐UTR of *YTHDF2* to exert its regulatory function. Overexpression of miR‐6125 in CRC cells did not affect the level of *YTHDF2* mRNA by affecting its stability (Figure 3E and Figure S1B, C), *YTHDF2* mRNA levels were analysed in 41 pairs of matched clinical samples from the TCGA database, and were also confirmed by 150 pairs of clinical samples we collected. The results indicated that compared with ANT, the level of YTHDF2 mRNA in tumour tissues was not significantly increased (Figure 3F and G); however, IHC staining showed that YTHDF2 protein levels were notable upregulated in CRC tissues compared with ANT (Figure 7A and B). In colorectal tumour tissues classified according to the largest tumour diameter, compared with the < 5 cm group, the expression of YTHDF2 was significantly upregulated in the ≥5 cm group (Figure 3H and I). The results of soft agar, ATP, CCK8 and EdU assays showed that YTHDF2 overexpression in SW480 and RKO cells promoted the proliferation of CRC cells significantly (Figure 3J–N and Figure S1B–G), indicating that YTHDF2 may play an oncogenic role in CRC. YTHDF2 was ectopically expressed in SW480 (miR‐6125) and RKO (miR‐6125) cells, and transfection efficacy was analysed by western blotting (Figure 3O). The results of soft agar, ATP, CCK8 and EdU assays indicated that miR‐6125 inhibited the proliferation of CRC cells by downregulating YTHDF2 expression (Figure 3P–S and Figure S1H–M).

### 2.4 Ectopic expression of miR‐6125 induces cell cycle arrest of human CRC cells at G0/G1 phase by downregulating Cyclin D1

**FIGURE 4:**
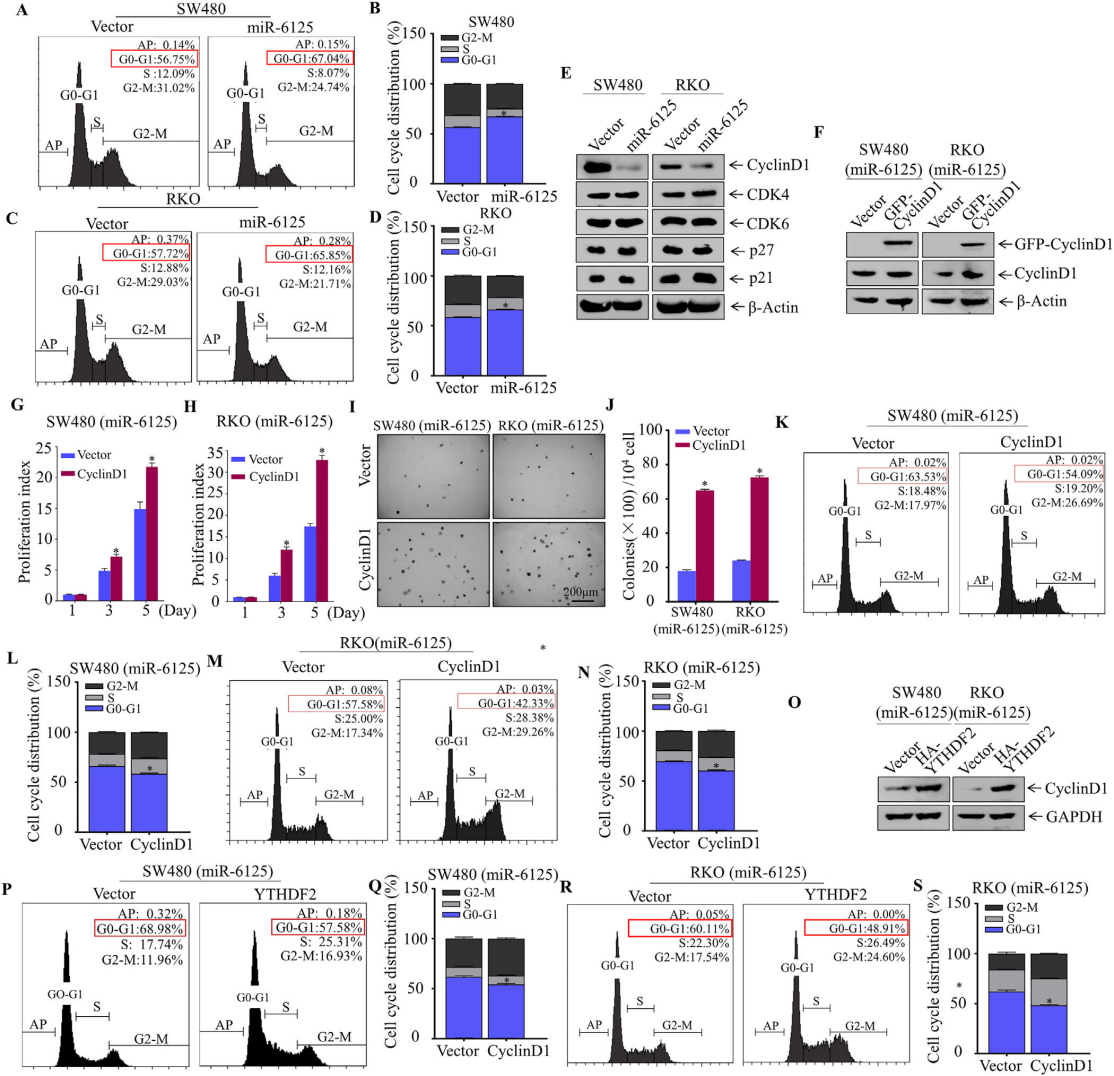
Ectopic expression of miR‐6125 induces cell cycle arrest at G0/G1 phase by downregulating Cyclin D1. (A–D) Effect of miR‐6125 overexpression on the cell cycle of SW480 and RKO cells, as detected by flow cytometry. (E) Western blot analysis of the expression of the cell cycle‐related proteins Cyclin D1, CDK4, CDK6, p27 and p21 in cells overexpressing miR‐6125, and in control cells. (F) GFP and GFP‐Cyclin D1 plasmids were stably transfected into SW480 (miR‐6125) and RKO (miR‐6125) cells, and transfection efficiency was detected by western blotting. (G–J) Effect of stable transformation of Cyclin D1 on SW480 (miR‐6125) and RKO (miR‐6125) cell anchorage‐independent growth and proliferation, as detected by soft agar and ATP assays. (K–N) After ectopic overexpression of Cyclin D1 in SW480 (miR‐6125) and RKO (miR‐6125) cells, flow cytometry was used to detect the changes of cell cycle. (O) After overexpression of YTHDF2 in SW480 (miR‐6125) and RKO (miR‐6125) cells, western blot was used to detect the expression level of Cyclin D1. (P–S) Flow cytometry was used to analyse cell cycle progression and the effect of stable transfection of YTHDF2 on cycle arrest induced by miR‐6125. An asterisk (*) indicates a significant difference at *p* < 0.05

Regulation of the cell cycle plays a vital role in malignant proliferation of tumours.^28^ Cell cycle progression is regulated by a series of cell cycle‐related proteins (Cyclins; CDKs; and CKIs), and alterations in expression of cyclins and related regulatory proteins are associated with progression of CRC.^29^ Therefore, we tested the influence of miR‐6125 on cell cycle in CRC cells. Overexpression of miR‐6125 in SW480 and RKO cells caused G0‐G1 phase arrest (Figure 4A–D). Detection of G0‐G1 phase regulatory proteins by western blotting indicated that Cyclin D1 was downregulated significantly in SW480 (miR‐6125) and RKO (miR‐6125) cells, whereas CDK4, CDK6, p21 and p27 were not affected significantly (Figure 4E). We found that knockdown of Cyclin D1 expression in SW480 cells (Figure S2A) significantly promoted the G0‐G1 phase arrest of SW480 cells (Figure S2B and C) and inhibited cell proliferation (Figure S2D–F). At the same time, Cyclin D1 was ectopically expressed in SW480 (miR‐6125) and RKO (miR‐6125) cells, and the transfection efficacy was tested by western blotting (Figure 4F). The results of soft agar, ATP, CCK8 and EdU assays and flow cytometry showed that miR‐6125‐mediated downregulation of Cyclin D1 caused cell cycle G0‐G1 arrest, thereby inhibiting the proliferation of CRC cells (Figure 4G–N and Figure S2G–L). Overexpression of YTHDF2 in SW480 (miR‐6125) and RKO (miR‐6125) cells upregulated Cyclin D1 expression significantly (Figure 4O) and inhibited the ability of miR‐6125 to block the cell cycle at the G0‐G1 phase (Figure 4P–S). These experiments demonstrated that miR‐6125 downregulates YTHDF2 and inhibits the growth of CRC by downregulating Cyclin D1 and causing cell cycle arrest.

### 2.5 Regulation of Cyclin D1 expression by miR‐6125 depends on YTHDF2‐meidated activation of the Wnt/β‐catenin pathway

**FIGURE 5:**
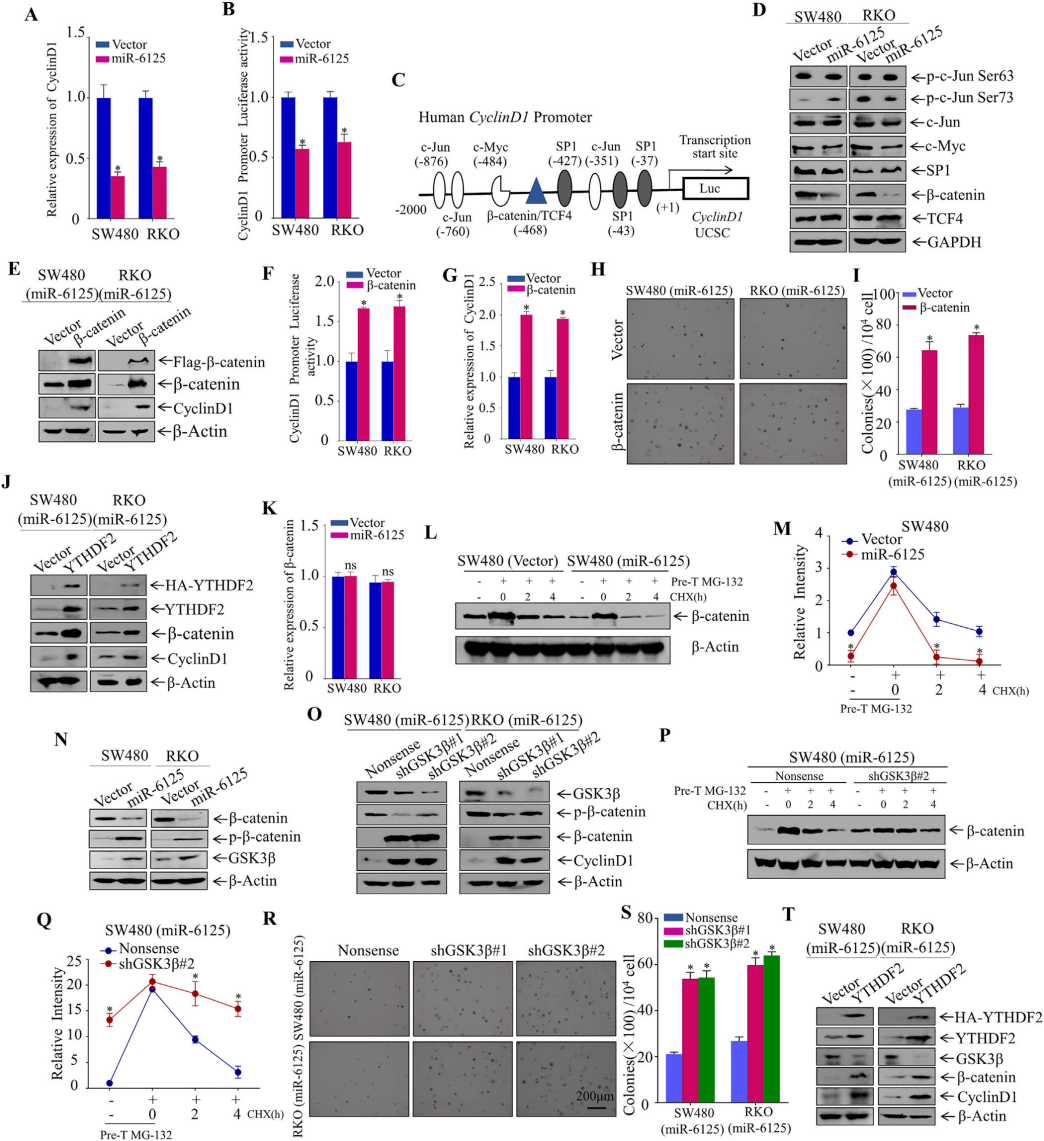
miR‐6125 regulates the expression of Cyclin D1 through the Wnt/β‐catenin pathway. (A) QPCR analysis of *Cyclin D1* expression in SW480 and RKO cells stably overexpressing miR‐6125 relative to that in control cells. (B) Dual luciferase activity of the *Cyclin D1* promoter in SW480, RKO and control cells stably overexpressing miR‐6125. (C) Bioinformatics method was used to analyse potential transcription factor binding sites in the promoter region of *Cyclin D1*. (D) Western blot analysis of transcription factors potentially affecting the expression of Cyclin D1 in SW480 and RKO cells stably overexpressing miR‐6125. (E) Flag‐β‐catenin and control plasmid were transfected into SW480 (miR‐6125) and RKO (miR‐6125) cells to establish a stable cell line. Transfection efficiency was analysed by western blotting and expression of Cyclin D1 was detected. (F and G) Dual luciferase activity of the *Cyclin D1* promoter in SW480 (miR‐6125), SW480 (miR‐6125/β‐catenin), RKO (miR‐6125) and RKO (miR‐6125/β‐catenin) cells, and qPCR detection of *Cyclin D1* mRNA expression. (H and I) Effect of stable transformation of β‐catenin on the anchorage‐independent growth of SW480 (miR‐6125) and RKO (miR‐6125) cells, as detected by a soft agar assay. (J) Western blot analysis of the transfection efficiency of YTHDF2, and expression of β‐catenin and Cyclin D1 proteins. (K) QPCR analysis of *β‐catenin* mRNA expression. (L and M) Western blot analysis of the protein degradation rate of β‐catenin, and densitometric quantification. (N) Western blot analysis of p‐β‐catenin (Ser33/37Thr41), β‐catenin and GSK3β expression in miR‐6125‐overexpressing SW480 and RKO cells and control cells. (O) Western blot analysis of the knockdown efficiency of GSK3β, and expression of p‐β‐catenin (Ser33/37Thr41), β‐catenin and Cyclin D1. (P and Q) Western blot analysis of the protein degradation rate of β‐catenin after GSK3β knockdown, and densitometric quantification. (R and S) Soft agar cloning assay to examine the effect of GSK3β knockdown in SW480 (miR‐6125) and RKO (miR‐6125) cells. (T) Western blot analysis of the transfection efficiency of YTHDF2, and expression of β‐catenin, Cyclin D1 and GSK3β. An asterisk (*) indicates a significant difference at *p* < 0.05

There are no studies addressing the mechanism by which YTHDF2 upregulates the expression of Cyclin D1. To explore this, we first showed that overexpression of miR‐6125 downregulated the expression level of *Cyclin D1* mRNA significantly (Figure 5A). To determine whether miR‐6125 regulates Cyclin D1 expression at the transcriptional level, we performed dual‐fluorescence experiments, which showed that miR‐6125 overexpression decreased *Cyclin D1* promoter activity significantly (Figure 5B). This indicates that miR‐6125 may regulate *Cyclin D1* transcription. Bioinformatic analysis was used to identify potential transcription factors and their binding sites in the promoter region of *Cyclin D1*. (Figure 5C); the results were verified by western blotting. Overexpression of miR‐6125 in SW480 and RKO cells downregulated β‐catenin significantly, whereas it had no significant or consistent effect on expression of other transcriptional regulatory factors (Figure 5D). Abnormal accumulation of β‐catenin causes its translocation from the cytoplasm to the nucleus, where it regulates the transcription of downstream genes, such as *Cyclin D1*. We performed nuclear‐plasma separation experiments using Lamin A and GAPDH as nuclear and cytoplasmic controls, respectively, and showed that miR‐6125 decreased β‐catenin levels in the cytoplasm and nucleus of SW480 and RKO cells significantly (Figure S3A). β‐Catenin was overexpressed in SW480 (miR‐6125) and RKO (miR‐6125) cells (Figure 5E), and nucleocytoplasmic separation experiments showed that ectopic expression of β‐catenin increased the level of β‐catenin in the cytoplasm and nucleus of SW480 (miR‐6125) and RKO (miR‐6125) cells (Figure S3B). The results indicated that ectopic expression of β‐catenin increased the expression level, promoter activity and mRNA level of *Cyclin D1* significantly (Figure 5E–G). The results of soft agar, ATP, CCK8 and EdU assays showed that miR‐6125 inhibited the proliferation of CRC cells by downregulating β‐catenin (Figure 5H and I; Figure S3C–J). Ectopic expression of YTHDF2 in SW480 (miR‐6125) and RKO (miR‐6125) cells upregulated β‐catenin and Cyclin D1 significantly (Figure 5J), suggesting the involvement of YTHDF2 in the regulation of β‐catenin and Cyclin D1 expression. We next explored the mechanism by which miR‐6125 regulates the expression of β‐catenin. The results of qPCR showed that miR‐6125 overexpression did not affect the mRNA level of *β‐catenin* significantly (Figure 5K). β‐Catenin degradation is mediated by a complex mainly formed by Axin, GSK3β and APC. Axin and APC interact with GSK3β, which binds to β‐catenin and promotes its phosphorylation; phosphorylated β‐catenin is then recognized by a ubiquitin ligase and targeted for degradation.^30^ Overexpression of miR‐6125 increased the protein degradation rate of β‐catenin significantly (Figure 5L and M). Western blot analysis showed that ectopic expression of miR‐6125 upregulated GSK3β and p‐β‐catenin (Ser33/37Thr41), and downregulated β‐catenin significantly in SW480 and RKO cells (Figure 5N). To demonstrate that β‐catenin degradation is caused by GSK3β‐mediated phosphorylation, we knocked down GSK3β in SW480 (miR‐6125) and RKO (miR‐6125) cells. GSK3β knockdown downregulated p‐β‐catenin (Ser33/37Thr41) levels and upregulated β‐catenin and Cyclin D1 levels significantly (Figure 5O), and decreased the degradation rate of the β‐catenin protein significantly (Figure 5P and Q). Functional experiments showed that knockdown of GSK3β weakened the ability of miR‐6125 to inhibit the proliferation of SW480 cells and RKO cells (Figure 5R and S; Figure S3K–R). Ectopic expression of YTHDF2 in SW480 (miR‐6125) and RKO (miR‐6125) cells downregulated GSK3β significantly and upregulated Cyclin D1 and β‐catenin significantly (Figure 5T), indicating that downregulation of YTHDF2 by miR‐6125 modulates the GSK3β‐β‐catenin‐Cyclin D1 pathway to regulate the proliferation of CRC.

### 2.6 MiR‐6125 downregulation promotes YTHDF2‐dependent recognition of N6‐methyladenosine‐modified GSK3β in CRC cells

**FIGURE 6:**
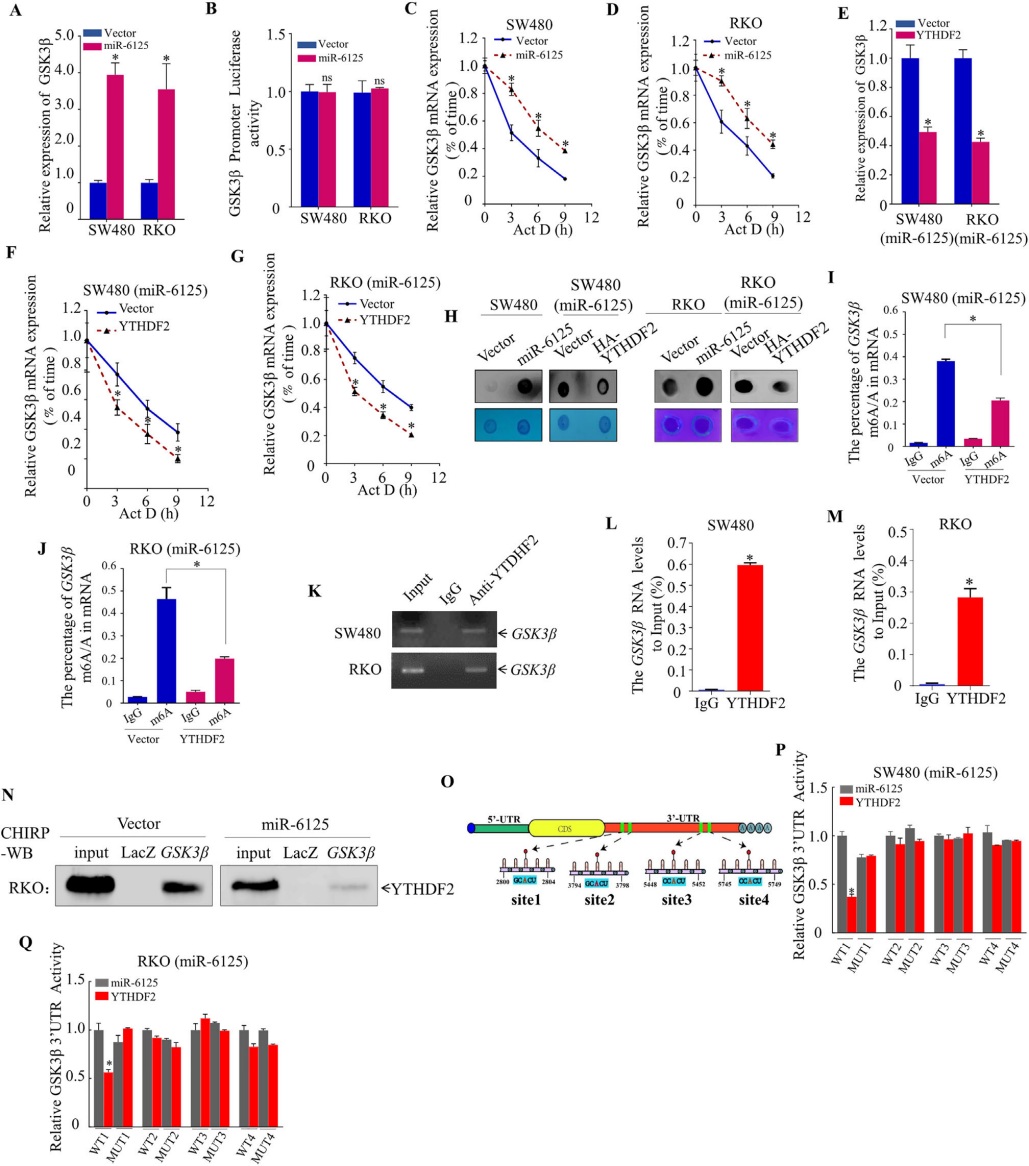
MiR‐6125 downregulation promotes YTHDF2‐dependent recognition of N6‐methyladenosine‐modified GSK3β in CRC cells. (A) QPCR detection of *GSK3β* mRNA expression in SW480 and RKO cells stably overexpressing miR‐6125 relative to that in control cells. (B) Dual luciferase activity of the *GSK3β* promoter in SW480 and RKO cells stably overexpressing miR‐6125. (C and D) QPCR to examine the stability of *GSK3β* mRNA in SW480 and RKO cells stably overexpressing miR‐6125. (E) QPCR detection of *GSK3β* mRNA expression in SW480 (miR‐6125) and RKO (miR‐6125) cells ectopically expressing YTHDF2. (F and G) QPCR detection of the effect of YTHDF2 on stability of *GSK3β* mRNA. (H) m6A dot blot assay detects the overall modification level of m6A in the cell lines. (I and J) MeRIP‐qPCR analysis of m6A‐modified *GSK3β* mRNA. (K) Nucleic acid electrophoresis was used to confirm that *GSK3β* mRNA is recognized by YTHDF2. (L and M) RIP‐qPCR analysis of *GSK3β* mRNA recognition by YTHDF2. (N) CHIRP‐WB assay was performed to detect the binding effect between *GSK3β* and YTHDF2 in RKO cells. (O) Bioinformatics method prediction of the *GSK3β* m6A modification site. (P and Q) Dual‐fluorescence reporter assay of *GSK3β* 3′‐UTR activity to verify that *GSK3β* mRNA can be identified and degraded at m6A modification sites. An asterisk (*) indicates a significant difference at *p* < 0.05

To elucidate the mechanism by which miR‐6125 regulates GSK3β, the mRNA level of *GSK3β* was measured. MiR‐6125 upregulated *GSK3β* mRNA significantly (Figure 6A), whereas it had no observable effect on the promoter activity of *GSK3β* (Figure 6B). In addition, miR‐6125 increased the stability of *GSK3β* mRNA significantly (Figure 6C and D). Ectopic expression of YTHDF2 in SW480 (miR‐6125) and RKO (miR‐6125) cells downregulated *GSK3β* mRNA significantly, and decreased its stability (Figure 6E–G). YTHDF2 recognizes and targets m6A‐modified RNA for degradation, thereby reducing the stability of the target RNA. The results of m6A dot blots showed that the overall level of m6A modification in SW480 (miR‐6125) and RKO (miR‐6125) cells was significantly higher than that in SW480 (Vector) and RKO (Vector) cells. At the same time, after reversing the expression of YTHDF2 in SW480 (miR‐6125) and RKO (miR‐6125) cells, the overall level of m6A modification was significantly downregulated, indicating that miR‐6125 changes the RNA m6A modification level of SW480 and RKO in CRC cells and the process is dependent on the expression change of YTHDF2 (Figure 6H). Methylated RNA immunoprecipitation (MeRIP)‐qPCR identified m6A modification sites on *GSK3β* mRNA, and overexpression of YTHDF2 decreased m6A‐modified *GSK3β* mRNA levels significantly (Figure 6I and J). The results of nucleic acid gel electrophoresis and RIP‐qPCR showed that YTHDF2 binds to *GSK3β* mRNA (Figure 6K–M), confirming that the m6A modification site in *GSK3β* mRNA is recognized by YTHDF2. We further verified that *GSK3β* mRNA can bind to YTHDF2 protein in RKO cells by chromatin isolation by RNA purification (CHIRP)‐WB assay (Figure 6N). To identify the specific modification sites, we first used bioinformatics methods to predict the m6A methylation site in *GSK3β* mRNA (Figure 6O). Double fluorescence experiments showed that after overexpressing YTHDF2 in the first group (WT1 and MUT1, note: WT1 means 2800–2804 site WT and MUT1 means 2800–2804 site MUT) of SW480 (miR‐6125) and RKO (miR‐6125) cells, it inhibited the activity of the *GSK3β* 3′‐UTR in the WT group significantly, but not in the MUT group (Figure 6P and Q). These results indicate that the 2800–2804 site of *GSK3β* is the main m6A modification site, and that YTHDF2 binding to this site targets the mRNA for degradation.

### 2.7 Correlation between miR‐6125, YTHDF2, GSK3β, β‐catenin and Cyclin D1 expression in CRC clinical tissues and nude mouse tissues

**FIGURE 7:**
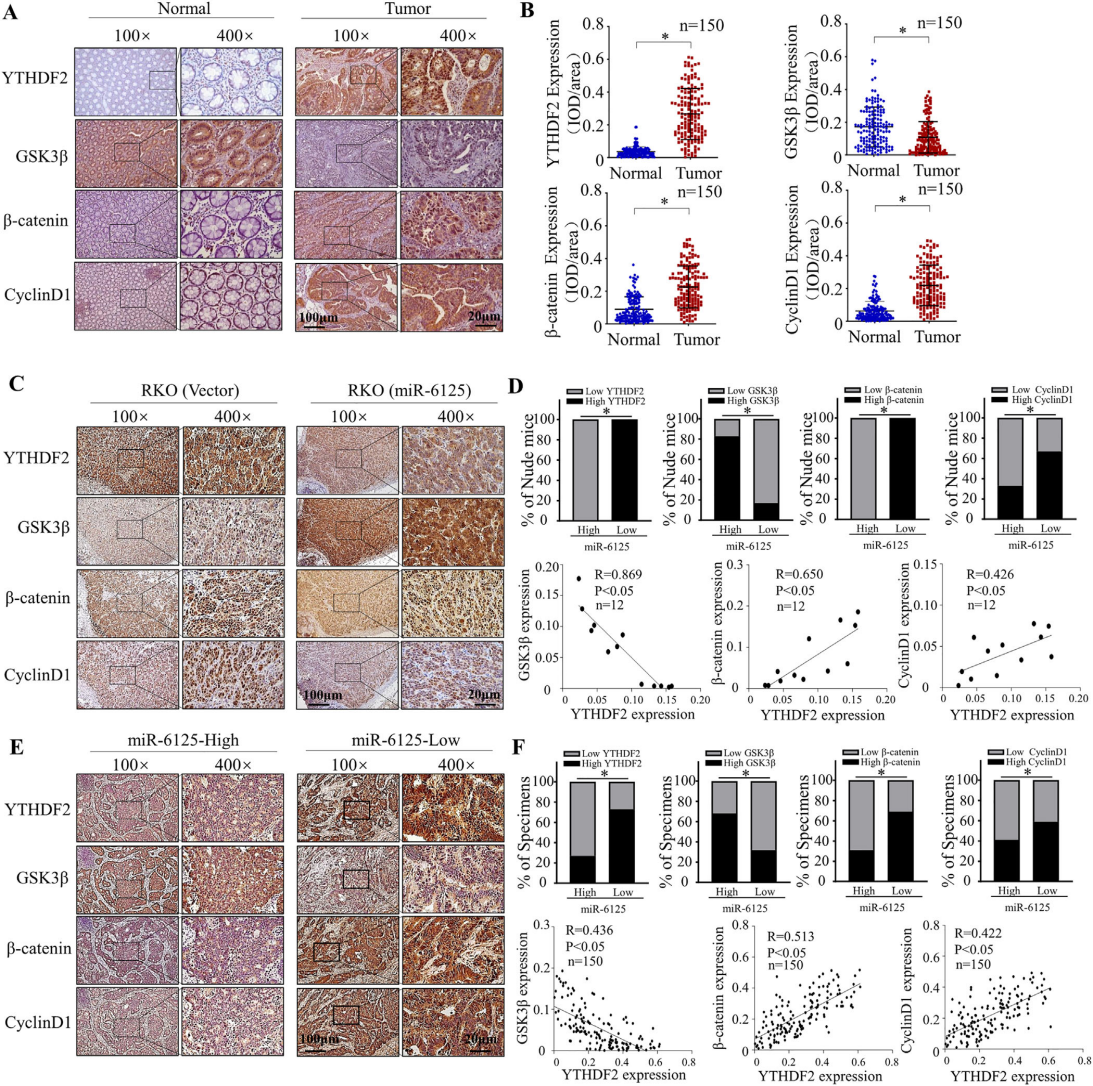
Correlation between miR‐6125, YTHDF2, GSK3β, β‐catenin and Cyclin D1 in CRC clinical tissues and nude mouse xenograft tumours. (A and B) Immunohistochemical (IHC) detection of miR‐6125, YTHDF2, GSK3β, β‐catenin and Cyclin D1 in 150 pairs of clinical tissues. (C and D) QPCR and IHC detection of miR‐6125, YTHDF2, GSK3β, β‐catenin and Cyclin D1 in six pairs nude mouse subcutaneous tumour samples. (E and F) QPCR and IHC detection of miR‐6125, YTHDF2, GSK3β, β‐catenin and Cyclin D1 in 150 of clinical tumour tissues. An asterisk (*) indicates a significant difference at *p* < 0.05

The above results showed that overexpression of miR‐6125 in SW480 and RKO cells with relatively low expression of miR‐6125 can inhibit YTHDF2‐GSK3β‐β‐catenin‐Cyclin D1 pathway, and further inhibited the proliferation of CRC cells. In order to verify the universality of this regulatory pathway, we used mirRNA inhibitor to inhibit the activity of miR‐6125 in HT29 cells with relatively high expression of miR‐6125, and preliminarily detected the effect of miR‐6125 on HT29 cells. The results showed that inhibition of miR‐6125 activity stabilized the 3′‐UTR of YTHDF2 (Figure S4A) and significantly promoted the proliferation of HT29 cells (Figure S4B and C). At the same time, western blot showed that after inhibiting miR‐6125, the protein expression levels of YTHDF2, β‐catenin and Cyclin D1 increased significantly, while the expression level of GSK3β decreased significantly (Figure S4D). We further verified the expression levels of YTHDF2, GSK3β, β‐catenin and Cyclin D1 in 150 pairs of tumour and ANTs. The results showed that the protein levels of YTHDF2, β‐catenin and Cyclin D1 were significantly upregulated in CRC tissues compared with ANT, whereas GSK3β protein level was higher in ANT than in CRC tissues (Figure 7A and B). Analysis of the correlation between miR‐6125, YTHDF2, GSK3β, β‐catenin and Cyclin D1 expression in a subcutaneous nude mouse xenograft model and in 150 paired clinical specimens revealed a positive correlation between miR‐6125 and GSK3β expression in clinical samples, whereas the expression levels of Cyclin D1, β‐catenin and YTHDF2 were positively correlated (Figure 7C–F). This indicates that the miR‐6125‐YTHDF2‐GSK3β‐β‐catenin‐Cyclin D1 regulatory pathway is important for progression of CRC.

### 2.8 Mechanistic diagram of miR‐6125‐mediated inhibition of YTHDF2‐GSK3β‐β‐catenin‐Cyclin D1 signalling pathway

**FIGURE 8:**
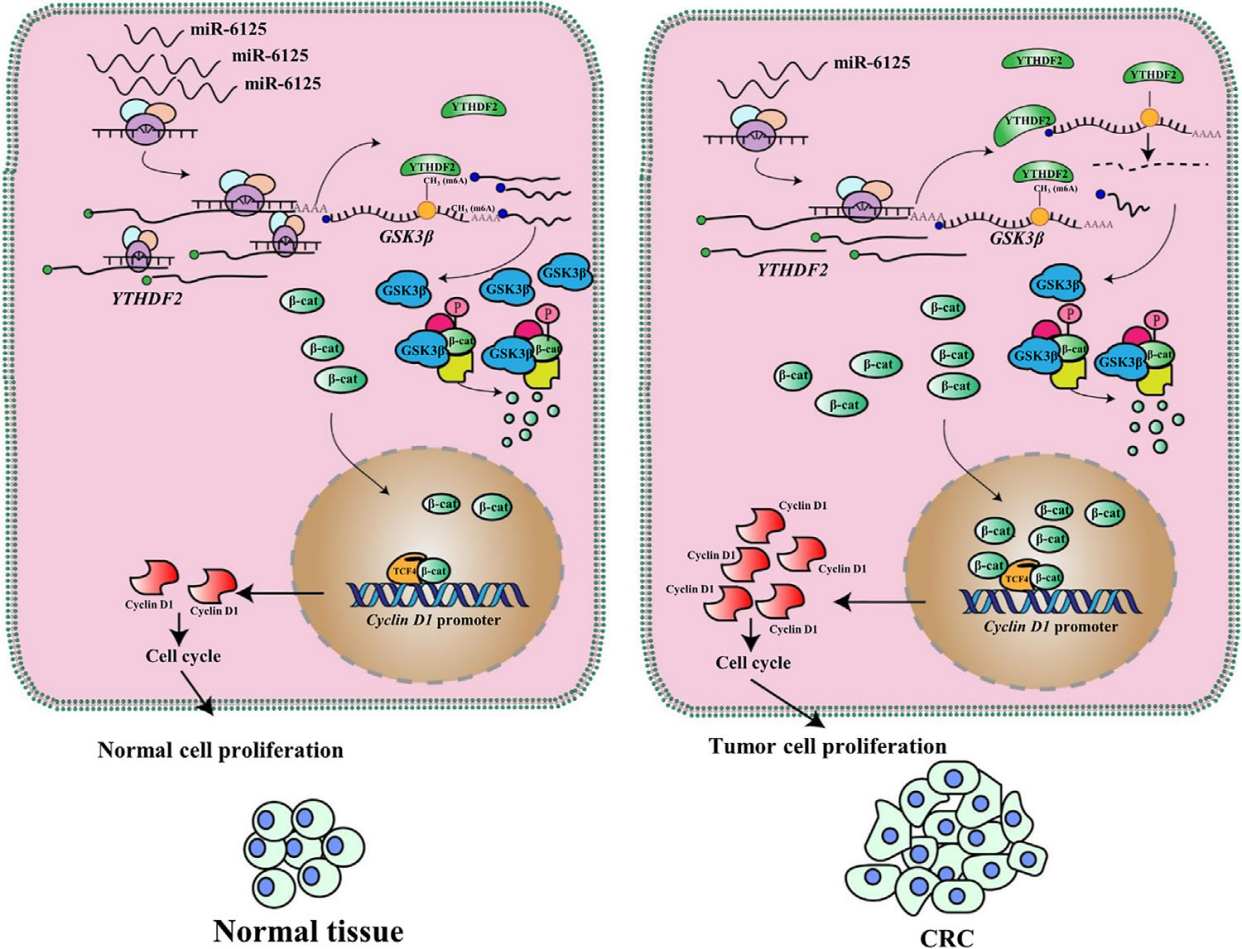
Diagram showing the effect of miR‐6125 on the YTHDF2‐GSK3β‐β‐catenin‐Cyclin D1 signalling pathway

The findings of the study are summarized in the mechanistic diagram shown in Figure 8. Briefly, miR‐6125 is downregulated in CRC tissues and cells, which attenuates the inhibition of *YTHDF2* mRNA translation, thereby increasing YTHDF2 protein levels. The increase in the YTHDF2 protein levels promotes the recognition of m6A‐modified *GSK3β* mRNA by YTHDF2. This decreases the stability of *GSK3β* mRNA, GSK3β protein levels, and the phosphorylation level of β‐catenin. Inhibition of protein degradation leads to abnormal accumulation of β‐catenin. Increased β‐catenin activates transcription of the downstream gene *Cyclin D1* and promotes cell cycle progression from G0 to G1, ultimately promoting malignant proliferation of CRC cells and CRC progression.

## 3 DISCUSSION

MiRNAs are conserved among species and show a high degree of sequence homology, suggesting that they play an important biological role.^9^ The complex and extensive regulatory effects of miRNAs make them promising therapeutic targets in many diseases. Compared with traditional monoclonal antibodies and small‐molecule compounds, miRNA‐based drugs have the advantages of easy design and synthesis and long‐lasting activity. This is particularly important for protein targets that are not druggable or when targeted drugs have poor efficacy, as well as when mutations result in drug resistance. In these cases, miRNA‐based drugs have become a reasonable and effective alternative. Many miRNA‐based drugs have been approved for marketing, and several miRNA drugs targeting tumours are in the clinical trial stage and have shown satisfactory results.^10^, ^31^, ^32^, ^33^, ^34^ Drug research and development based on miRNAs, as well as the combined application of miRNA‐based drugs and traditional targeted drugs, provide innovative new therapeutic approaches to cancer. Although miRNAs play a key role in the progression of tumours, including CRC, and despite their potential clinical application, many miRNAs remain to be identified, and their functions and mechanisms need to be elucidated. This limits our understanding of the progression of CRC, as well as the development and utilization of related drugs. This study showed that miR‐6125 is downregulated significantly in CRC, and that its expression correlates negatively with tumour size and poor prognosis. These findings indicate that miR‐6125 can be used as a diagnostic marker for CRC. It also serves as a prognostic marker as its expression is closely related to tumour progression. Functional and mechanistic experiments showed that miR‐6125 significantly inhibited the proliferation of CRC cells by regulating the YTHDF2‐GSK3β‐β‐catenin‐Cyclin D1 pathway. This study is the first to prove that miR‐6125 acts as a CRC suppressor and that it has important biological functions, thereby providing new insight into the progression of CRC and suggesting that miR‐6125 is a potential therapeutic target.

The m6A methylation process is mediated by three types of enzyme; namely, writers responsible for methylation, such as methyltransferase‐like 3/14 (METTL3/14), WT1‐associated protein (WTAP) and KIAA1429; erasers responsible for demethylation, such as AlkB homolog 5 (ALKBH5) and fat mass and obesity‐associated Protein (FTO); and proteins that recognize methylated RNA readers, such as YTH domain‐containing 1/2 (YTHDC1/2) and YTH domain‐containing family protein 1/2/3 (YTHDF1/2/3).^35^ Abnormal RNA methylation is closely related to the progression of tumours. In CRC, METTL14 degrades m6A‐modified SRY‐related HMG‐box 4 (*SOX4*) mRNA through a YTHDF2‐dependent pathway, thereby inhibiting the metastasis of CRC.^36^ METTL14 can also degrade the m6A‐modified long non‐coding RNA (lncRNA) XIST through a YTHDF2‐dependent pathway, thereby inhibiting the proliferation and metastasis of CRC.^37^ Although YTHDF2 plays a key role in CRC progression, no studies have explored the protein expression level of YTHDF2 in CRC, or regulation of its expression. In this study, we show for the first time that miR‐6125 targets the 3′‐UTR of *YTHDF2* and downregulates its expression. We found that YTHDF2 is expressed at high levels in CRC, and its expression is positively correlated with tumour growth. Overexpression of YTHDF2 promoted proliferation of CRC cells. Mechanistic experiments showed that YTHDF2 recognized m6A‐modified *GSK3β* mRNA and targeted it for degradation. This caused β‐catenin protein accumulation, activated the Wnt‐β‐catenin pathway and promoted the proliferation of CRC cells. This mechanism differs from those reported previously, including the degradation of SOX4 by YTHDF2 and the role of the lncRNA XIST and other proto‐oncogenes in inhibiting the progression of CRC. During the development of adipocytes, YTHDF2 recognizes and degrades m6A‐modified *Cyclin D1* mRNA, downregulating the Cyclin D1 protein and leading to cell cycle arrest, thereby inhibiting fat synthesis.^38^ In the present study, we found that YTHDF2 upregulated Cyclin D1 and promoted CRC cell cycle progression, which is not consistent with previous findings and reflects both the versatility of YTHDF2 and the tissue specificity of its function. Taken together with previous findings, the present study improves our understanding of the function of YTHDF2, as well as the cooperative relationship between RNA methylation genes. This provides valuable insight into development of drugs targeting RNA methylase‐related genes. This may also be useful for development, utilization and evaluation of the safety and effectiveness of clinical drugs. The mechanisms regulating RNA methylation are complex, and the relationship between YTHDF2 and the occurrence and development of CRC requires additional research.

The Wnt pathway plays a vital role in CRC. Abnormal activation of the Wnt pathway is an important mechanism underlying the development of CRC.^24^ The role of the classic Wnt/β‐catenin pathway in CRC has been studied extensively. A complex composed of APC, Axin and GSK3β in the cytoplasm promotes the phosphorylation of β‐catenin by GSK3β. β‐Transducing repeat‐containing protein (β‐Trcp) and other E3 ligases recognize phosphorylated β‐catenin and target it for degradation. During this process, inactivation of GSK3β causes β‐catenin to accumulate in the cell and translocate to the nucleus,^25^ where it binds to T‐cell factor/lymphoid enhancer‐binding factor 4 (TCF4) to activate the downstream target genes *Cyclin D1*, matrix metallopeptidase 7 *(MMP7*) and *c‐Myc* to promote the occurrence and development of tumours.^39^, ^40^, ^41^ Targeted drugs based on the Wnt/β‐catenin pathway have clinical application value and are potential strategies for the treatment of CRC. However, despite the development of Wnt/β‐catenin signalling inhibitors to treat CRC,^42^, ^43^, ^44^ there are currently no approved molecular therapies that target Wnt/β‐catenin signalling in clinical practice. The complexity of Wnt/β‐catenin pathway regulation limits the development and application of targeted drugs. For example, although GSK3β plays an important role in Wnt/β‐catenin signalling, the mechanisms underlying regulation of GSK3β expression remain unclear. In previous studies, GSK3β had been proved that play both inhibitory and promoting effects on the progression of CRC.^45^, ^46^, ^47^, ^48^ In this study, we showed that GSK3β acts as a tumour suppressor gene; m6A‐modifed GSK3β was recognized and degraded by YTHDF2, leading to the activation of Wnt/β‐catenin signalling and further promoting the proliferation of CRC cells in vitro and in vivo. These results indicate that methylation of *GSK3β* mRNA is an important mechanism regulating GSK3β expression and Wnt/β‐catenin signalling, and that it may be a key molecular event affecting the progression of CRC. This study provides a valuable reference for the development and utilization of CRC‐targeted drugs based on Wnt/β‐catenin pathway.

In conclusion, we showed that expression of miR‐6125 was downregulated significantly and correlated negatively with a poor prognosis for CRC. Downregulation of miR‐6125 affected the expression of YTHDF2‐GSK3β‐β‐catenin‐Cyclin D1 pathway‐related proteins, promoting cell cycle progression from G0 to G1 phase and proliferation of CRC cells. These findings indicate that miR‐6125 and YTHDF2 are potential targets for the clinical treatment of CRC. Further research is necessary to elucidate the roles of miR‐6125 and YTHDF2 in CRC and in other biological processes.

## 4 MATERIALS AND METHODS

### 4.1 Plasmids, reagents and antibodies

The miR‐6125 precursor overexpression plasmid (HmiR1561‐MR03) and its control plasmid were purchased from Genecopoeia (Guangzhou Science Park, Guangzhou, China). The miR‐6125 micrOFFTM inhibitor (S1127) and its control reagent (U0709) were purchased from RIBOBIO Company (Guangzhou, China). YTHDF2, β‐catenin overexpression plasmid, shGSK3β plasmid and control plasmids were constructed by Miaoling Company (Wuhan, China), and the GFP‐Cyclin D1 plasmid was constructed in the laboratory. Cycloheximide was obtained from Calbiochem (San Diego, CA, USA). Antibodies against Cyclin D1 (2968), c‐Jun (9165), p‐c‐Jun (Ser63) (2361), p‐c‐Jun (Ser73) (3270), SP1 (9389), HA (3724), p‐β‐catenin (Ser33/37Thr41) (9561), GSK3β (12456), Flag (14793) and β‐catenin (8480) were purchased from CST (Boston, MA, USA). Antibodies against GFP (SC‐9996), CDK4 (SC‐260), p21 (SC‐397), p27 (SC‐1641), CDK6 (SC‐7961) and c‐Myc (SC‐764) were obtained from Santa Cruz Biotechnology (Dallas, TX, USA). Antibodies against GAPDH (Ab0037) and β‐Actin (Ab0011) were purchased from Abways Technology (Shanghai, China). Antibodies against YTHDF2 (24744‐1‐AP) were purchased from Proteintech (Wuhan, China).

### 4.2 Human samples and cell lines

One hundred and fifty pairs of CRC tissue and adjacent normal human tissues were collected from the First Affiliated Hospital of Wenzhou Medical University. HCT116 (CBP60028 COBIOER, Nanjing, China), HT29 cells (CBP60011 COBIOER), RKO (CBP60006 COBIOER), SW480 (CCL‐228; ATCC, Rockefeller, MD, USA), CCD 841 CoN (CRL‐1790, ATCC) and CCD18‐Co (CRL‐1459, ATCC) cells were cultured in medium recommended by ATCC.

### 4.3 Real‐time PCR

PCR steps have been described in detail in our previous studies.^49^ PCR amplification was performed using primers specific for the following genes: human *GSK3β* (forward: 5′‐TGT CAA GTA ATC CAC CTC TGG C‐3′, reverse: 5′‐TTA GCA TCT GAC GCT GT‐3′), human *β‐catenin* (forward: 5′‐ AGG TCT GAG CAG CTT CA‐3′, reverse: 5′‐ TTC AAA TAC CCT CAG GGG AAC A‐3′), human *Cyclin D1* (forward: 5′‐ GCT GCG AAG TGG AAA CCA TC‐3′, reverse: 5′‐ CCT TCT GCA CAC ATT TGA AA‐3′), human *YTHDF2* (forward: 5′‐CCT TAG GTG GAG CCA TGA TTG‐3′, reverse: 5′‐TCT GTG CTA CCC AAC TTC AGT‐3′), human *GAPDH* (forward: 5′‐GAC TCA TGA CCA CAG TCC ATG C‐3′, reverse: 5′‐CAG GTC AGG TCC ACC ACC ACT GA‐3′) and miR‐6125 (forward: 5′‐GCG GAA GGC GGA GCG GA‐3′).

### 4.4 Western blot

The protein concentration of the sample was measured by NanoDrop One (Thermo Fisher Scientific). Protein samples were separated by SDS‐PAGE (120 V, 90 min), then the protein on SDS‐PAGE was transferred to PVDF membranes. Membranes were incubated with corresponding antibody at 4°C for more than 12 h, then the second antibody was incubated for 2.5 h. The ECF developer (RPN5785, GE Healthcare, Boston, MA, USA) was diluted with TBS in proper proportion, and the membrane was scanned on the GE Healthcare.

### 4.5 Nude mouse xenograft model

Female BALB/c athymic nude mice were obtained from GemPharmatech (Nanjing, Jiangsu, China; license number: SCXK 2018‐0008). At age of 4–5 weeks, nude mice were randomly divided into four groups and injected subcutaneously with SW480 (Vector), SW480 (miR‐6125), RKO (Vector) or RKO (miR‐6125) cells on the back region (5.0 × 10^6^ cells in 100 μl PBS/mouse). After 3–4 weeks, the mice were euthanized, the tumours were removed by operation, and the tumours were photographed and weighed according to the groups.

### 4.6 Immunohistochemistry

Paraffin‐embedded tissues were sliced into 4–5 μm sections using a microtome, samples were dewaxed and hydrated with xylene and alcohol rinsed with PBS, and subjected to high‐pressure antigen recovery with citrate buffer in a microwave oven (100°C for 4×7 min, when finished, cool the sample to room temperature). The tissue samples were incubated with 3% H_2_O_2_ for about 10 min followed by blocking with 3% FBS for 30–60 min. The tissue was incubated with the corresponding antibody at 4°C for at least 12 h. For IHC staining, antibodies specific for Cyclin D1 (Cell Signaling Technology, 2968), MKI67 (Abcam, ab16667), GSK3β (Cell Signaling Technology, 12456T), β‐catenin (Cell Signaling Technology, 8480P) and YTHDF2 (Proteintech, 24744‐1‐AP) were used.

### 4.7 RIP‐qPCR and MeRIP‐qPCR

RIP experiments were performed with RNA Immunoprecipitation Kit (BersinBio, bes5101, Guangzhou, China). Four micrograms of YTHDF2 (24744‐1‐AP), m6A (Cell Signaling Technology, 56593) and IgG control antibodies were used for RIP analysis. After cell lysis, 1.8 ml of lysate was obtained, and 0.1 ml of lysate was used as input. The remaining lysate was divided into two parts and immunoprecipitated with YTHDF2, m6A and IgG control antibodies to obtain *GSK3β*‐enriched fragments, and *GSK3β* enrichment was detected by qRT‐PCR. The primers used to detect the *GSK3β* m6A enrichment region were as follows: forward: 5′‐AAT TGG TTG GGA GCT TAG CAG G‐3′, and reverse: 5′‐TCC CTG GGT TAC GAA TGA TAC AC‐3′.

### 4.8 EdU assay

Cells in the logarithmic growth phase were analysed using an EdU Assay Kit (Ribobio, c10310‐2). After EDU labelling, paraformaldehyde fixation, Apollo staining and DNA staining, images were taken under a fluorescence microscope.

### 4.9 M6A dot blot assay

The cells were lysed with Qiagen reagent or Trizol reagent, and total RNA was extracted. After the sufficient mix of 5 μl RNA samples, 15 μl formaldehyde/SSC buffer (10× SSC contains 6.15 mol/L formaldehyde) (Sigma‐Aldrich, Saint Louis, MO, USA) and RNA incubation buffer, draw a circle on the NC membrane with a hydrophobic pen to prevent droplet dispersion, then spot 5–8 μl of pre‐treated RNA sample on the NC membrane. Upon drying, the RNA on the membrane was cross‐linked in ultraviolet cross linker. The crosslinked membrane was incubated with 0.02% methylene blue (Sigma‐Aldrich) for 5–10 min. Upon washing 5 min with TBST, the stained membrane was photographed and then incubated with the antibody of anti‐m6A (Cell Signaling Technology, 56593) overnight at 4℃. Then the second antibody was incubated for 2.5 h. The ECF developer was diluted with TBS in proper proportion, and the membrane was scanned on the GE Healthcare.

### 4.10 Chromatin isolation by RNA purification assay

ChIRP assay was performed by ChIRP RNA Interactome Kit (BersinBio, Bes5104, Guangzhou, China) according to the manufacturer's instructions. Specific recognition of GSK3β Biotin probes for RNA was designed and synthesized by Gzscbio Company (Guangzhou, China). The protein binding to *GSK3β* was detected by western blot (the steps were the same as described in the 4.4 method above).

### 4.11 Statistical analysis

Experimental data are expressed as the mean ± standard deviation, and the data were processed and plotted with graphpad prism 5.0. Log‐rank test was performed to analyse the difference of survival rate between the two groups, Student's *t* test was performed to evaluate the difference between two groups, *p* ≤ 0.05 means there is a significant difference compared with the control group.

## COMPETING INTERESTS

The authors declare that they have no competing interests.

## Supporting information


**Figure S1**. The *YTHDF2* 3′‐UTR mutation site is shown (A). The effect of overexpression of miR‐6125 on the stability of *YTHDF2* mRNA was detected in SW480 and RKO cells (B and C). The effect of YTHDF2 overexpression on proliferation of SW480 (vector) and RKO (vector) cells (D–I) and SW480 (miR‐6125) and RKO (miR‐6125) cells (J–M) was analysed using CCK8 and EdU assays.

**Figure S2**. Western blot was performed to detect Cyclin D1 knockdown efficiency in SW480 cells (A). Flow cytometry was performed to detect the effect of knockdown of Cyclin D1 on the cell cycle of SW480 cells (B and C). Soft agar and ATP assays were performed to detect the effect of knockdown of Cyclin D1 on the proliferation of SW480 cells. The effect of Cyclin D1 on proliferation of SW480 (miR‐6125) and RKO (miR‐6125) cells was analysed in CCK8 and EdU assays (G–L).

**Figure S3**. Western blot analysis of the effect of miR‐6125 on the nuclear and cytoplasmic distribution of β‐catenin in SW480 and RKO cells (A). Western blot analysis was used to detect the distribution of β‐catenin in the cytoplasm and nucleus of SW480 (miR‐6125) and RKO (miR‐6125) cells expressing β‐catenin (B). β‐Catenin was ectopically expressed in SW480 (miR‐6125) and RKO (miR‐6125) cells and the effect on cell proliferation was analysed in CCK8 and EdU assays (C–J). The effect of GSK3β knockdown on cell proliferation in SW480 (miR‐6125) and RKO (miR‐6125) cells was detected by CCK8 and EdU assays (K–R).

**Figure S4**. After inhibiting the activity of miR‐6125 in HT29 cells, the effect on YTHDF2 3′‐UTR activity was detected (A). After inhibiting the activity of miR‐6125 in HT29 cells, the effect on the proliferation of HT29 cells was detected by ATP assay and soft agar assay (B and C). After inhibiting the activity of miR‐6125 in HT29 cells, western blot was used to detect the changes of protein expression level of related molecules (D).

**Table S1**. Information about CRC patients including case number and overall survival (OS).

## Data Availability

The data that support the findings of this study are available from the corresponding author upon reasonable request.
