# Corrigendum

**DOI:** 10.1002/ctm2.1085

**Published:** 2022-10-17

In the originally published article by Hongyan Li et al. (2021)^1^, the authors regret the error in supplementary figure panel: The image of the western blot of Lamin A in Supplementary Figure S3B had been wrongly placed.

This error has now been corrected in new figure and do not affect the results or conclusions of this work. The authors would like to apologize for any inconvenience caused.

## Supporting information


**Figure S3**. Western blot analysis was used to detect the distribution of β‐catenin in the cytoplasm and nucleus of SW480 (miR‐6125) and RKO (miR‐6125) cells expressing β‐catenin (B).

## References

[1] Li, H. , Zhang, N. , Jiao, X. , et al. Downregulation of microRNA‐6125 promotes colorectal cancer growth through YTHDF2‐dependent recognition of N6‐methyladenosine‐modified GSK3β. Clin Transl Med. 2021;11:e602. 10.1002/ctm2.602 34709763 PMC8516342

