# Supplementary figures and images for: Downregulation of microRNA‐6125 promotes colorectal cancer growth through YTHDF2‐dependent recognition of N6‐methyladenosine‐modified GSK3β
Source: Clin Transl Med. 2021 Oct 14;11(10):e602. doi: 10.1002/ctm2.602 (PMC8516342; doi:10.1002/ctm2.602)

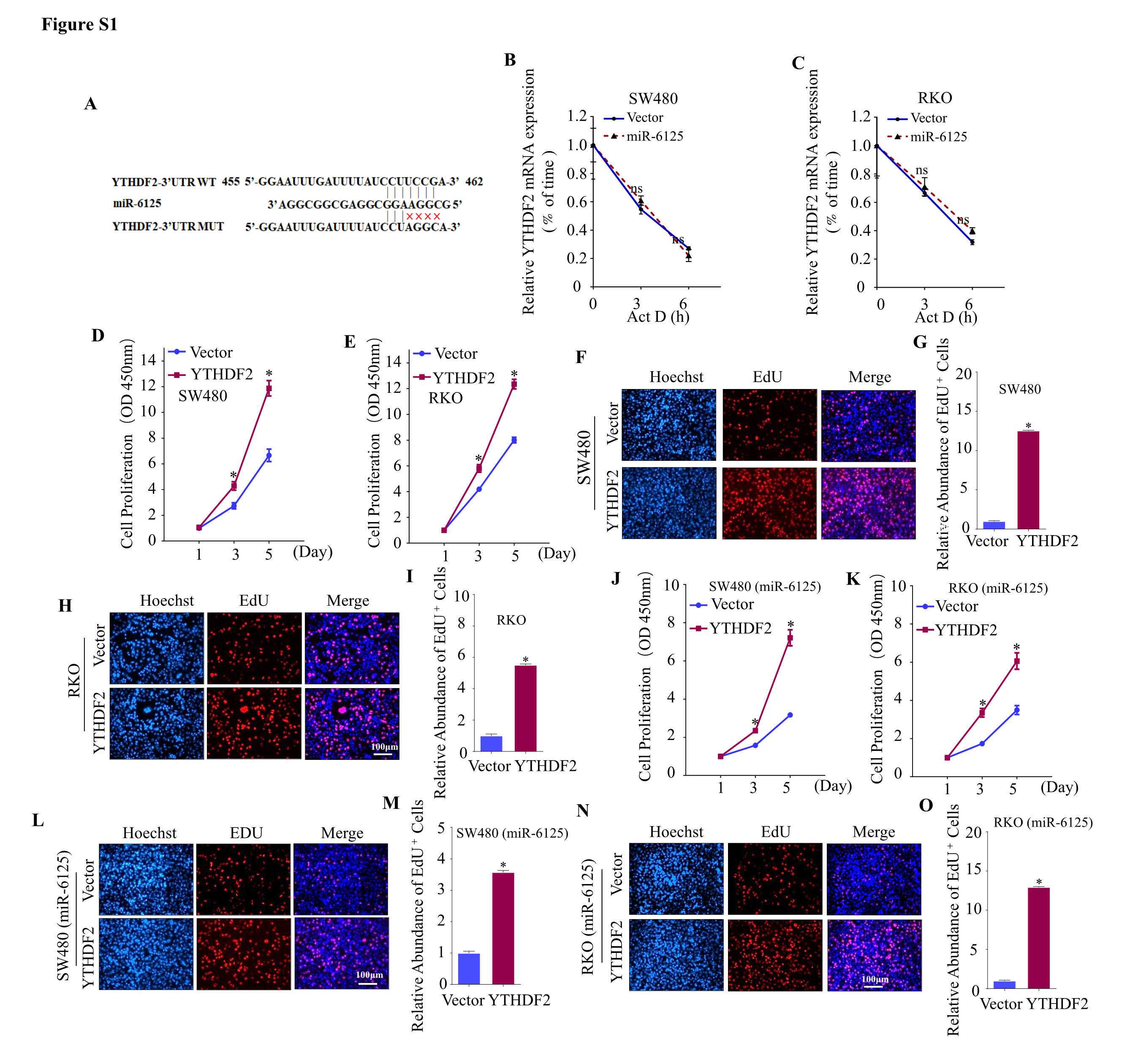

Supplement: Supplementary file 1 — Figure S1. The YTHDF2 3′‐UTR mutation site is shown (A). The effect of overexpression of miR‐6125 on the stability of YTHDF2 mRNA was detected in SW480 and RKO cells (B and C). The effect of YTHDF2 overexpression on proliferation of SW480 (vector) and RKO (vector) cells (D–I) and SW480 (miR‐6125) and RKO (miR‐6125) cells (J–M) was analysed using CCK8 and EdU assays. [file CTM2-11-e602-s003.tiff]

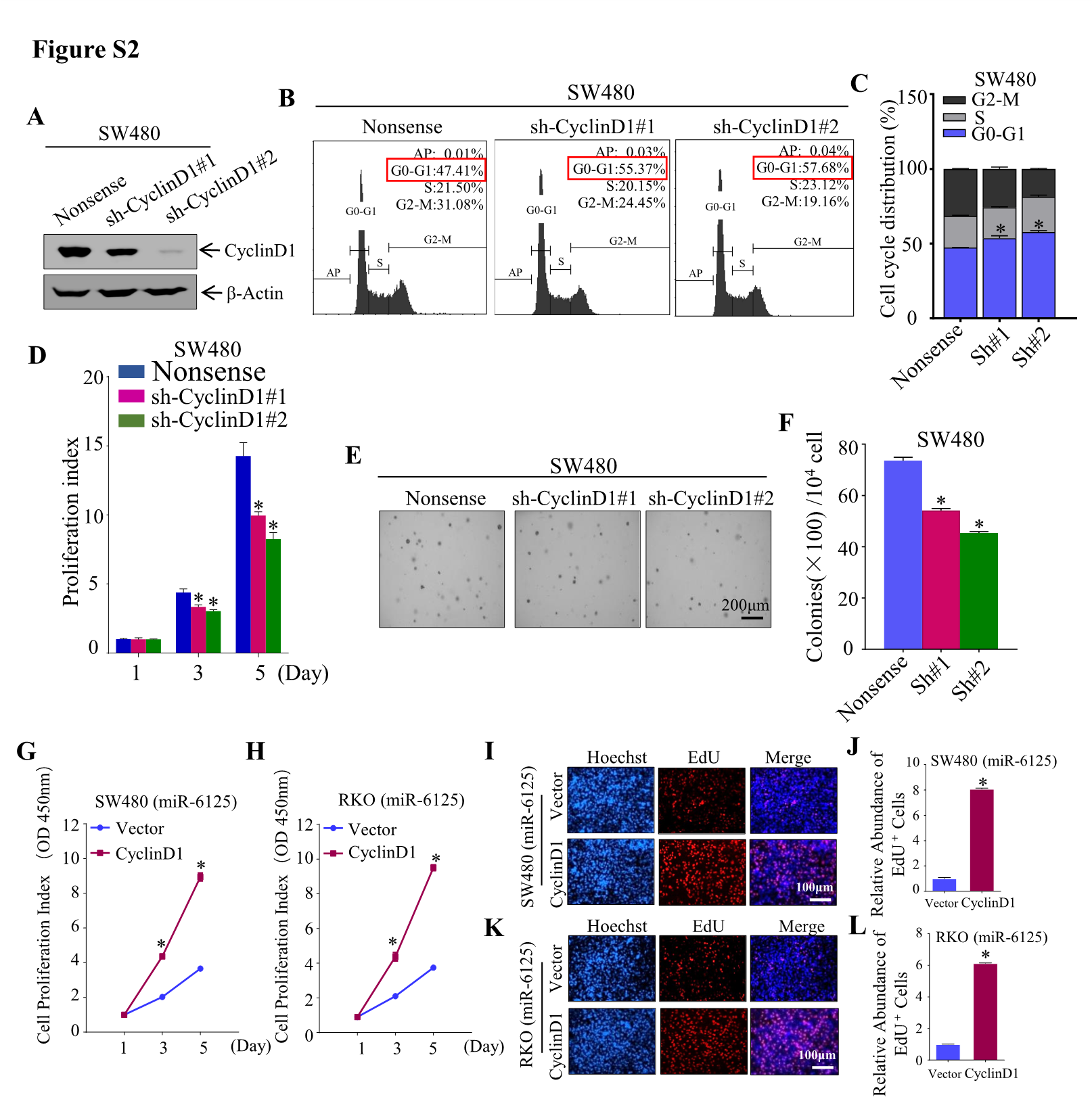

Supplement: Supplementary file 2 — Figure S2. Western blot was performed to detect Cyclin D1 knockdown efficiency in SW480 cells (A). Flow cytometry was performed to detect the effect of knockdown of Cyclin D1 on the cell cycle of SW480 cells (B and C). Soft agar and ATP assays were performed to detect the effect of knockdown of Cyclin D1 on the proliferation of SW480 cells. The effect of Cyclin D1 on proliferation of SW480 (miR‐6125) and RKO (miR‐6125) cells was analysed in CCK8 and EdU assays (G–L). [file CTM2-11-e602-s005.tiff]

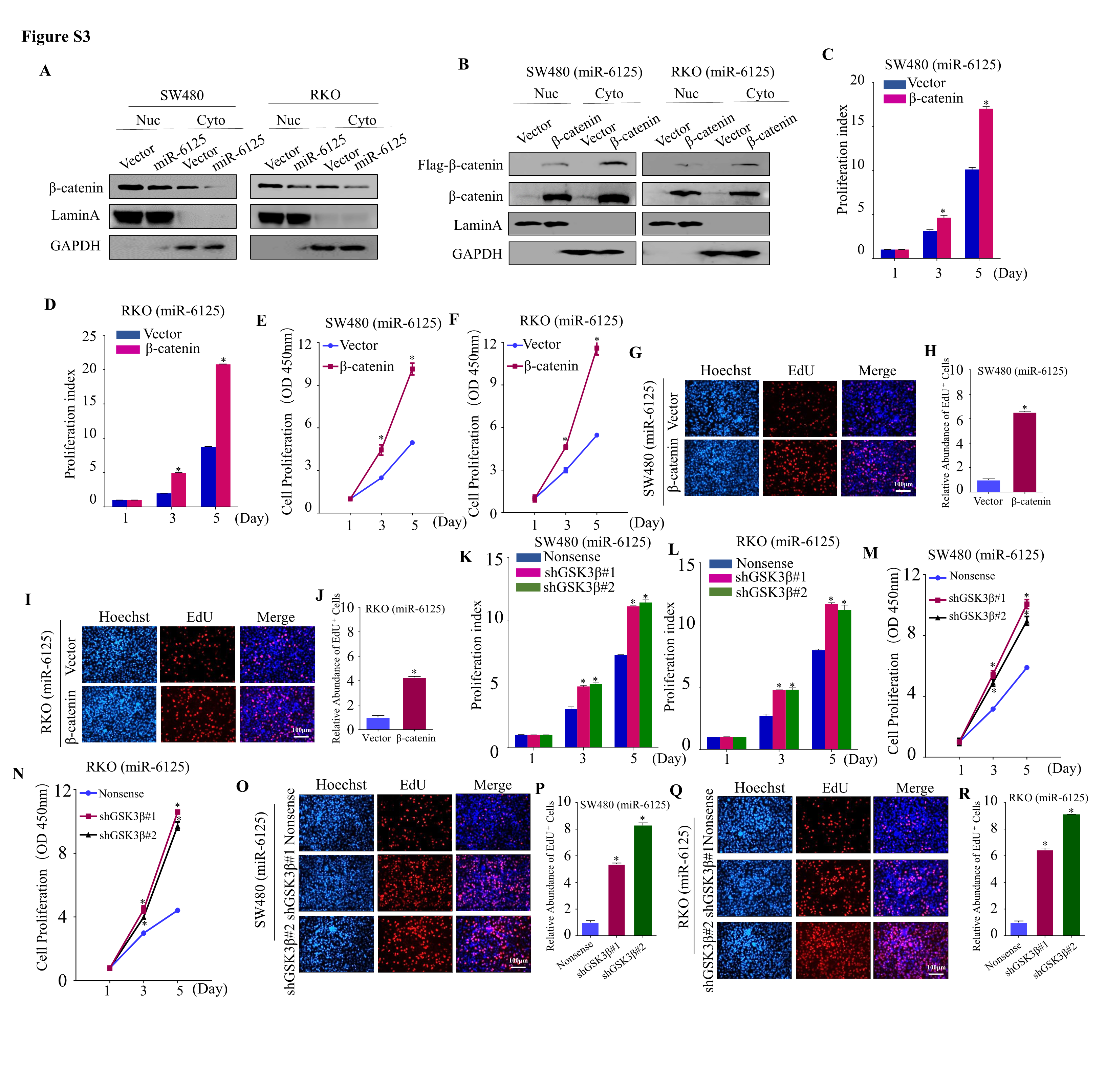

Supplement: Supplementary file 3 — Figure S3. Western blot analysis of the effect of miR‐6125 on the nuclear and cytoplasmic distribution of β‐catenin in SW480 and RKO cells (A). Western blot analysis was used to detect the distribution of β‐catenin in the cytoplasm and nucleus of SW480 (miR‐6125) and RKO (miR‐6125) cells expressing β‐catenin (B). β‐Catenin was ectopically expressed in SW480 (miR‐6125) and RKO (miR‐6125) cells and the effect on cell proliferation was analysed in CCK8 and EdU assays (C–J). The effect of GSK3β knockdown on cell proliferation in SW480 (miR‐6125) and RKO (miR‐6125) cells was detected by CCK8 and EdU assays (K–R). [file CTM2-11-e602-s001.tiff]

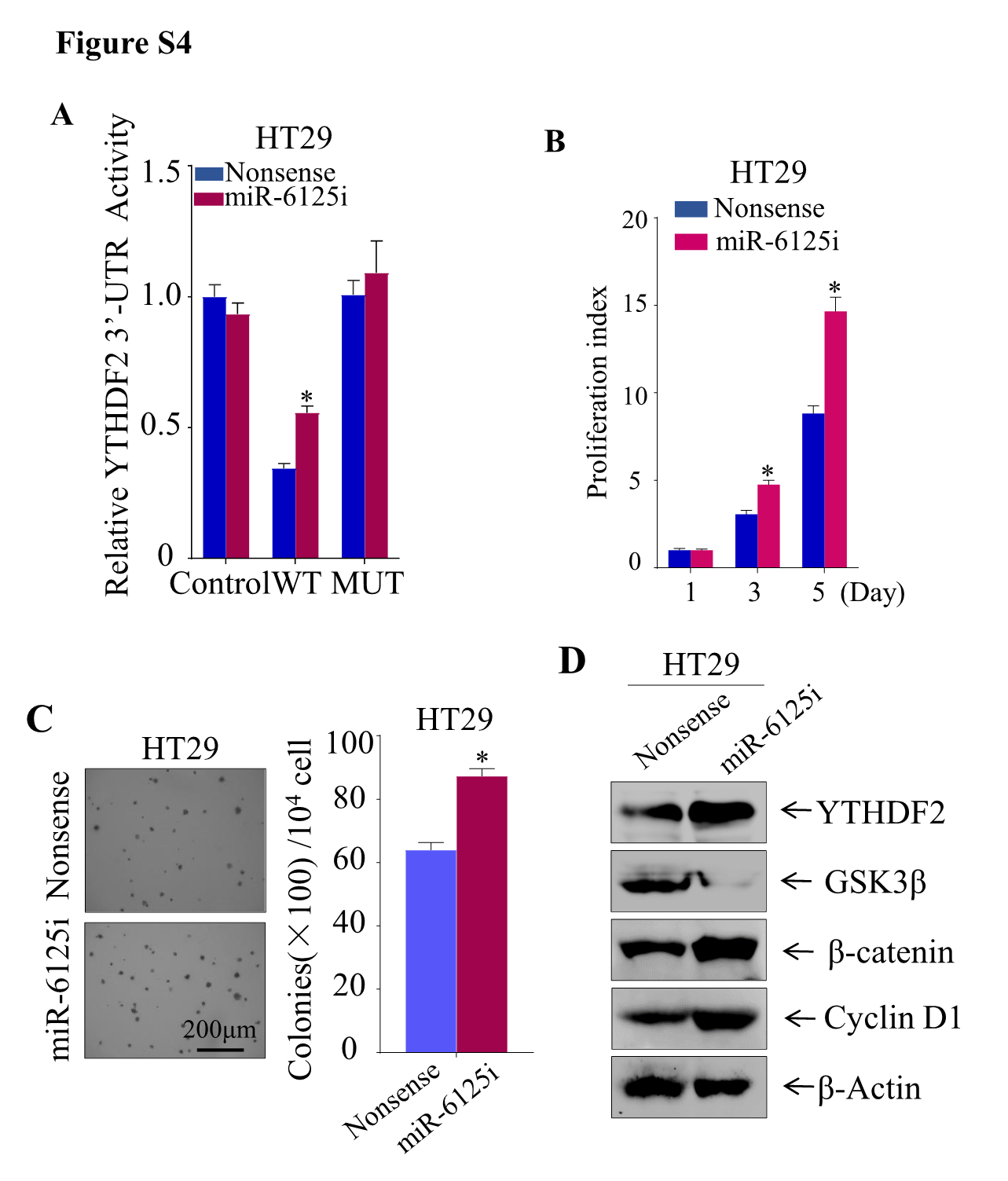

Supplement: Supplementary file 4 — Figure S4. After inhibiting the activity of miR‐6125 in HT29 cells, the effect on YTHDF2 3′‐UTR activity was detected (A). After inhibiting the activity of miR‐6125 in HT29 cells, the effect on the proliferation of HT29 cells was detected by ATP assay and soft agar assay (B and C). After inhibiting the activity of miR‐6125 in HT29 cells, western blot was used to detect the changes of protein expression level of related molecules (D). [file CTM2-11-e602-s002.tiff]

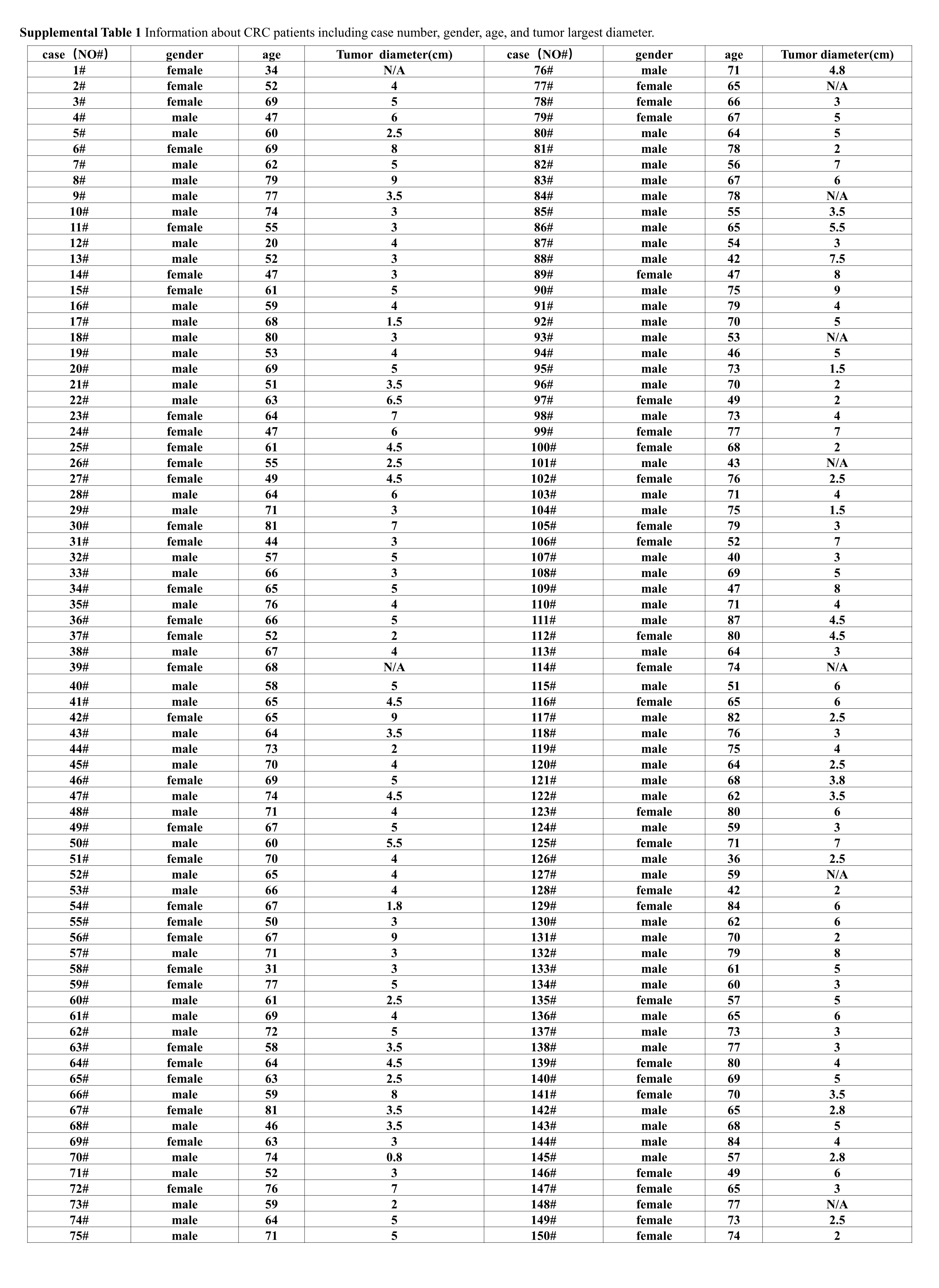

Supplement: Supplementary file 5 — Table S1. Information about CRC patients including case number and overall survival (OS). [file CTM2-11-e602-s004.tiff]
