# Supplementary figures and images for: Corrigendum
Source: Clin Transl Med. 2022 Oct 17;12(10):e1085. doi: 10.1002/ctm2.1085 (PMC9574599; doi:10.1002/ctm2.1085)

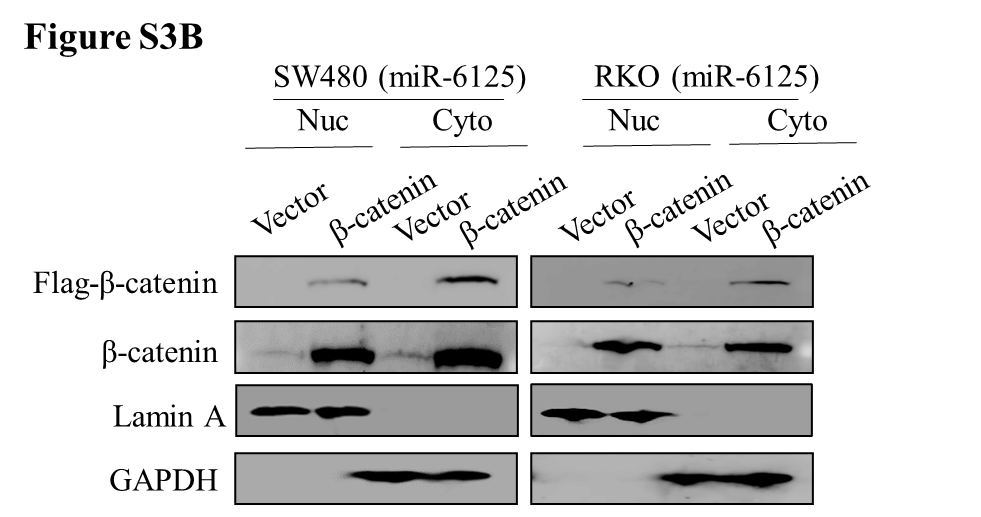

Supplement: Supplementary file 1 — Figure S3. Western blot analysis was used to detect the distribution of β‐catenin in the cytoplasm and nucleus of SW480 (miR‐6125) and RKO (miR‐6125) cells expressing β‐catenin (B). [file CTM2-12-e1085-s001.docx]
